# FGF19 and FGF21 for the Treatment of NASH—Two Sides of the Same Coin? Differential and Overlapping Effects of FGF19 and FGF21 From Mice to Human

**DOI:** 10.3389/fendo.2020.601349

**Published:** 2020-12-22

**Authors:** Emma Henriksson, Birgitte Andersen

**Affiliations:** Liver Disease Research, Global Drug Discovery, Novo Nordisk A/S, Maaloev, Denmark

**Keywords:** NAFLD, NASH, FGF19, FGF21, triglycerides, cholesterol, bile acids, adiponectin

## Abstract

FGF19 and FGF21 analogues are currently in clinical development for the potential treatment of NASH. In Phase 2 clinical trials analogues of FGF19 and FGF21 decrease hepatic steatosis with up to 70% (MRI-PDFF) after 12 weeks and as early as 12–16 weeks of treatment an improvement in NASH resolution and fibrosis has been observed. Therefore, this class of compounds is currently of great interest in the field of NASH. FGF19 and FGF21 belong to the endocrine FGF19 subfamily and both require the co-receptor beta-klotho for binding and signalling through the FGF receptors. FGF19 is expressed in the ileal enterocytes and is released into the enterohepatic circulation in response to bile acids stimuli and in the liver FGF19 inhibits hepatic bile acids synthesis by transcriptional regulation of Cyp7A1, which is the rate limiting enzyme. FGF21 is, on the other hand, highly expressed in the liver and is released in response to high glucose, high free-fatty acids and low amino-acid supply and regulates energy, glucose and lipid homeostasis by actions in the CNS and in the adipose tissue. FGF19 and FGF21 are differentially expressed, have distinct target tissues and separate physiological functions. It is therefore of peculiar interest to understand why treatment with both FGF19 and FGF21 analogues have strong beneficial effects on NASH parameters in mice and human and whether the mode of action is overlapping This review will highlight the physiological and pharmacological effects of FGF19 and FGF21. The potential mode of action behind the anti-steatotic, anti-inflammatory and anti-fibrotic effects of FGF19 and FGF21 will be discussed. Finally, development of drugs is always a risk benefit analysis and the human relevance of adverse effects observed in pre-clinical species as well as findings in humans will be discussed. The aim is to provide a comprehensive overview of the current understanding of this drug class for the potential treatment of NASH.

## Introduction

### NAFLD

Non-alcoholic fatty liver disease (NAFLD) is a spectrum of liver disease ranging from simple steatosis to non-alcoholic steatohepatitis (NASH) and cirrhosis. NAFLD is the most common chronic liver disorder in Western countries and in the USA 30% of the adult population suffers from NAFLD (1). Simple steatosis can if not treated progress to NASH, which is defined by the presence of steatosis, lobular inflammation, cellular ballooning and varying degrees of fibrosis (2). Eventually, NASH can lead to cirrhosis and hepatocellular carcinoma (HCC) (3) and NASH is expected to be the leading cause of liver transplantations by 2030 (4). Obesity is associated with an increased risk of NAFLD and the risk is increasing with increasing BMI (4). Furthermore, the risk of NASH is increased 2–3-fold in patients with Type 2 Diabetes (T2D). It is also well established that genetic factors predispose individuals to NAFLD and around 25% of people diagnosed with NAFLD have polymorphisms in adiponutrin (PNPLA3) (5, 6). Patients with NASH have an overall higher mortality rate compared to age-matched controls and the primary cause of death in the early stages of NASH is cardiovascular diseases, while the cause of death in patients with late stage fibrosis is liver related (7). There is currently no treatment for NASH (8) and with the discouraging outlook of the amount of future liver transplantations, there is a large unmet medical need to identify and develop effective treatment options for the benefit of the patients.

**Figure 1:**
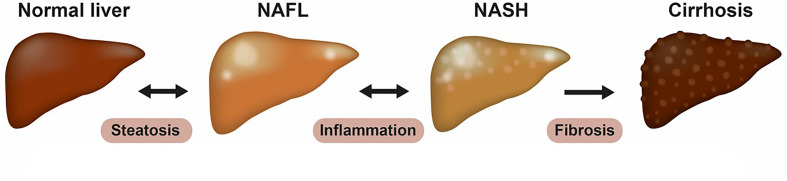
The spectrum of NAFLD. Non-alcoholic fatty liver disease (NAFLD) is a spectrum of diseases, now recognized as the most common liver disease worldwide. It ranges from simple steatosis (NAFL) to non-alcoholic steatohepatitis (NASH), cirrhosis, and its complications. NAFL is defined as the presence of at least 5% hepatic steatosis without evidence of hepatocellular injury. NASH is characterized histologically by presence of steatosis (> 5%), lobular inflammation and ballooning hepatocytes, and can be present with or without fibrosis. Fibrosis is graded from 0–4 based on histological appearance, where stage 4 often is referred to as cirrhosis and can be further divided into compensated or de-compensated cirrhosis.

NAFLD is an integral component of the complex metabolic disturbances observed in patients with type 2 diabetes and obesity (7) and hepatic steatosis is an imbalance between free fatty acid (FFA) influx, FFA utilization and very low-density lipoprotein (VLDL) secretion. In addition, the *de novo* lipogenesis (DNL) is increased secondary to hyperinsulinemia and an excessive intake of simple sugars. The increasing quantity of fructose in the Western diet may therefore be a major contributor to the development of NAFLD (5, 7). The insult caused by lipid accumulation in the hepatocyte induces mitochondrial dysfunction, oxidative stress, dysregulated apoptosis, activation of proinflammatory cytokines and profibrogenic factors, which in turn active the hepatic stellate cell and cause fibrosis. Furthermore, dysregulation of adipokines (like low adiponectin) (6) and increase in gut-derived proinflammatory signals such as lipopolysaccharides (LPS) by microbiota (9) may also contribute to the development of NASH. The spectrum of NAFLD is shown in **Figure 1**.

### Treatment for NASH

No treatment has been approved for NASH and non-pharmacologic treatment of NAFLD/NASH aiming to reduce fatty liver by body weight (BW) loss and exercise is recommended. This is often found to be challenging for the majority and experimental therapies may be initiated with insulin-sensitizing agents (pioglitazone) and anti-oxidative compounds (vitamin E) which demonstrate improvements in steatosis, inflammation and fibrosis to some extent (10). However, the effects of these therapies are not well established and safety concerns makes pioglitazone less attractive in clinical practise (11) leaving a large medical gap. Despite numerous drug candidates in clinical development for NASH (12) there has been a few setbacks in the field due failure of several clinical candidates [simtuzumab (13), selonsertib (14), elafibranor (15)] which were unable to show any significant effect on the resolution of NASH or lowering of fibrosis in phase 3, while treatment with obeticholic acid (OCA) led to a small but significant reduction in fibrosis (16). However, an accelerated approval has not been granted by the FDA as the observed efficacy of OCA potentially does not outweigh the potential risks. Many other compounds are in clinical development (17) but most of these have not yet reached phase 3. The drug candidates can on a top level be divided into three main categories 1) metabolic compounds with effect on steatosis (PPAR agonists, ACC inhibitors, Ketohexokinase inhibitors, DGAT2 inhibitors, SCD1 inhibitors, SGTL2 inhibitors, GLP-1 receptor agonist or derivates thereof, THRb agonists, FGF19, and FGF21 analogues etc.); 2) compounds that target inflammation (AOC3 inhibitors, CCR2/5 inhibitors, Gal3 inhibitors); and 3) compounds with anti-fibrotic effect (FXR agonist, FGF19, and FGF21 analogues, ASK-1 inhibitors, Loxl2 inhibitors).

By removing the insults (steatosis and inflammation) a positive effect on fibrosis is expected. It is well established that bariatric surgery resolves fibrosis over time (18, 19). Thus, the duration of the trials may be of critical importance to reach significant effect on fibrosis. It is therefore of great interest to note that both FGF19 and FGF21 analogues which often have been categorized as metabolic compounds, show NASH resolution and lower fibrosis in trials of relative short duration.

### The endocrine FGFs

FGF19 (FGF15 in rodents) and FGF21 belong to the FGF19 subfamily of endocrine FGFs based on their atypical structure. Members of this subfamily lack the heparin binding domain and have no or very low affinity for heparan sulphate (HS). This enables the endocrine FGFs to escape the cellular matrix and enter the circulation to act as hormonal-like messengers (20). FGF15/FGF19 and FGF21 cannot bind the FGF receptors without the presence of a non-signalling transmembrane co-receptor beta-klotho (KLB) (8, 21–24). In contrast to the FGF receptors, which are ubiquitously expressed (11), KLB expression is limited to a few tissues/cells including liver, gallbladder, exocrine pancreas, white adipose tissue (WAT), brown adipose tissue (BAT) and in very specific regions of the central nervous system (CNS), (suprachiasmatic nucleus/paraventricular nucleus in the hypothalamus and dorsal vagal complex of the hindbrain) (11, 25, 26). In the presence of KLB, FGF19, and FGF21 bind and signal through the short isoforms (c-isoform) of the FGFR 1, 2, and 3 (22, 23) while FGF19 also signals through FGFR4 (27). FGF21 has the highest affinity for the FGFR1c/KLB complex followed by the FGFR3c/KLB (22) while FGF19 has highest affinity for FGFR4/KLB followed by the FGFR1c/KBL (23, 28). FGF19 has, moreover, been reported to induce FGFR4 signalling in the absence of KLB but in presence of HS (26, 29, 30). KLB is co-expressed with FGFR1c in the CNS and in adipocytes, while FGFR4 and KLB are co-expressed in hepatocytes (11, 23). Thus, the primary target tissue of FGF21 is the CNS and the adipose tissue, while FGF19 acts on the hepatocytes.

#### FGF15/FGF19

The *FGF19* gene was cloned in 1999 by homology to the mouse orthologue *Fgf15* from retina (31, 32). The rodent FGF15 and human FGF19 are orthologues but only share 52% amino acid identity. FGF15/FGF19 is expressed in the ileal enterocytes (11) and is released into the enterohepatic circulation postprandially in response to bile acids *via* activation of the farnesoid X receptor (FXR) (33, 34). FGF19/FGF15 regulates hepatic bile acids synthesis by activation of the hepatic FGFR4/KLB complex which decreases the expression of the rate limiting enzyme [cholesterol 7 alpha-hydroxylase (CYP7A1)] in bile acids synthesis (32, 35, 36). However, hepatic Cyp7a1 expression is also regulated directly by FXR through the small heterodimer partner (SHP)-and the pregnane X receptor (PXR) (37–40). Bile acids are strong detergents and thus their synthesis is tightly regulated to prevent enterohepatic damage (41). FGF19/FGF15 also controls refilling of bile acids into the gall bladder after a meal (42, 43), and has been described to be a postprandial activator of hepatic protein and glycogen synthesis and to inhibit hepatic gluconeogenesis (42, 44). Global deletion of the *Fgf15 or Klb* in mice increases hepatic *Cyp7a1* mRNA expression, plasma bile acids and increases fecal bile acids excretion (32, 45).

FGF19 is also highly expressed in liver in HCC and has been suggested to be responsible for growth and invasion of tumors through its interactions with FGFR4/KLB (46). (46, 47) Furthermore, FGF15 knockout (ko) mice display an impairment in liver regeneration after partial hepatectomy (48) and FGF15 ko mice have less and smaller tumours and fewer histological neoplastic lesions in response to diethylnitrosamine-(DEN)-induced HCC compared to wild type-mice (49). Oppositely, FGF15 overexpressing transgenic (tg) mice have very low bile acids and an increase in hepatocyte proliferation suggesting that FGF15 plays a critical role in liver regeneration (50). Other authors, however, claim that in contrast to FGF19, FGF15 is not carcinogenic in several murine models (51) and that fundamental species-associated differences between FGF19 and FGF15 restrict the relevance of mouse models for the study of carcinogenic effect of the FXR/FGF19 pathway.

#### FGF21

**Figure 2:**
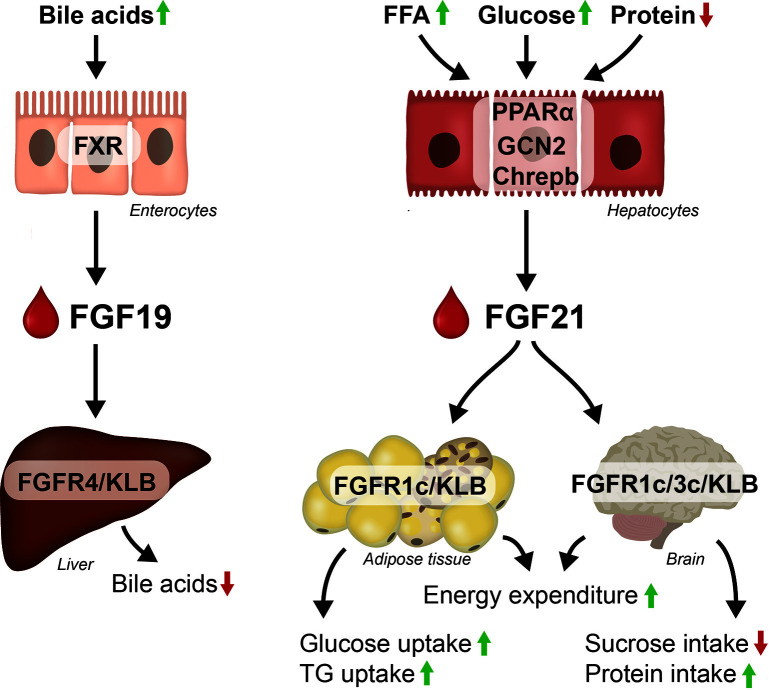
Regulation and effect on FGF19 and FGF21. FGF19 is released from the enterocytes in response to bile acids and suppresses bile acids synthesis in hepatocytes. FGF21 is mainly expressed in hepatocytes in response to FFA, glucose and lack of amino acids. FGF21 acts in the CNS and in the adipose tissue to control glucose, lipid and energy metabolism, by increasing glucose and TG uptake into the adipose tissue, by increasing EE and altering food preferences.

The mouse and human Fgf21/FGF21 genes were cloned by Nishimura et al. in 2000 (52). FGF21 is highly expressed in liver and pancreas while lower expression is observed in adipose tissue and skeletal muscle across species (11, 52–56). In mice, FGF21 is released from the liver in response to fasting and FFA (57, 58) by activation of peroxisome proliferator-activated receptors (PPAR)α receptor (59). The increase in plasma FGF21 in response to PPARα stimulation has been suggested to be involved in a negative feedback loop to inhibit lipolysis (60). Hepatic FGF21 is also increased in response to high glucose *via* activation of Carbohydrate-response element-binding protein (ChREBP) (61) and FGF21 lowers the preference for glucose intake (62) by stimulation of glutamatergic neurons in the ventromedial hypothalamus (VMH) (63). Moreover, FGF21 facilitates glucose (64), lipid uptake (65) and adipogenesis in the adipose tissue (66), which prevent ectopic lipid accumulation in liver and skeletal muscle (67). Importantly, FGF21 is also released in response to insufficient amino acid supply triggered by the integrated stress response which activates the general control nonderepressible 2 (GCN2) (68) and induces FGF21 transcription. In response to protein restriction FGF21 is required to increase food intake (in order to meet the protein demand) and energy expenditure (EE) (68). Moreover, FGF21-treated mice are hyperphagic to overcome the increase in EE and the mice prefer protein over carbohydrate (69, 70). The FGF21 ko mice (71–74) have decreased thermogenic ability (decreased BAT activity) (75, 76) and lack the ability to expand subcutaneous fat (77) potentially due to decreased PPARγ expression in the adipose tissue (71). The FGF21 ko mice are furthermore insulin resistant (78) and the glucose excursion rate in response to an intraperitoneal glucose tolerance test is increased (71). A simplified overview of the physiological role of FGF19 and FGF21 is shown in **Figure 2**.

### Overlapping and distinct effect of FGF19 and FGF21 in mice

As FGF19 is not expressed in rodents it is important to notice that FGF19 and FGF15 despite being orthologues display different mitogenic and metabolic functions in mice (28, 51) and interestingly the FGF19 overexpressing tg mice (79) display overlapping phenotype with the FGF21 tg mice. Both genetic models have increased EE, increased brown adipose tissue, decreased BW, lower fat mass, lower liver fat, and lower plasma IGF-1 (64, 76, 80). Also, treatment with FGF19 ameliorate the metabolic phenotype in high fat fed mice (81). However, unlike FGF19, FGF15 does not lower blood glucose (BG) in diabetic mice (28, 51) indicating that FGF19 display a differential receptor selectivity compared to FGF15. Notably, FGF15 does not activate down-stream signaling of the mouse FGFR1c/KLB receptor complex despite binding (28). The inability of FGF15 to lower BG in diabetic mice may be associated with the lack of FGFR1c/KLB activity (28, 51). This is in agreement with data showing that a FGFR4/KLB selective variant of FGF19 lowers bile acids and induces hepatic proliferation while it does not decrease BG in diabetic mice, while a FGFR1c selective FGF19 variant (FGF19dCTD) maintains the ability to lower BG but does not regulate bile acids in mice (24, 30).

The extracellular domain of KLB is approx. 80% conserved between mouse and human, but species differences have been observed and FGF15 does for example not bind human KLB (28). Oppositely, FGF19 and FGF21 bind mouse KLB with higher affinity than human KLB (28, 82). FGF19 is furthermore a potent activator of both FGFR4/mouse and human KLB complex (28) and is approximately 1000-fold more potent than FGF21 in inhibiting *CYP7A1* mRNA expression in primary human hepatocytes (28, 83, 84). FGF19 also binds FGFR1c/KLB complex and binds FGFR1c/mouse KLB with approx. 25 higher potency than the FGFR1c/human KLB (28). FGF21 binds the FGFR1c/human KLB complex with 2-fold higher affinity than FGF19. The ability of FGF19 to activate FGFR1c in presence of both mice and human KLB is of high importance in order to understand the metabolic actions of FGF19 in mice and humans as FGF19 therefore has overlapping effect with FGF21 which primarily use FGFR1c/KLB as its major receptor complex. However, in contrast to FGF21 which decreases plasma triglycerides (TG) and cholesterol (28, 85) FGF19 increases plasma TG and cholesterol (86, 87) in mice. As the FGFR4 selective FGF19 variant (FGF19dCTD) retains its ability to lower bile acids synthesis and increases plasma TG and total cholesterol levels the negative effects of FGF19 on plasma lipids is mediated by FGFR4/KLB activation (30). An overview of FGF15, FGF21, and FGF19 with respect to protein size, expression, order of receptors affinity, metabolic, and mitogenic effects is shown in **Table 1**.

**Table 1:** Overview of FGF15, FGF19 and FGF21.

	FGF15	FGF19	FGF21
Amino acids (mature)	218 aa	216 aa	181 aa
Mw	25.2 kDa	24 kDa	20 kDa
Co-receptor	KLB	KLB (HS)	KLB
Affinity	Mouse KLB	Mouse KLB>human KLB	Mouse KLB>human KLB
FGFR signaling	FGFR4	FGFR4>FGFR1c, FGFR2c, FGFR3c	FGFR1>FGFR3c>>FGFR2c>>FGFR4
Knock-in	Low bile acids levels	Increased EE, low BW, increased insulin sensitivity Low bile acids levels	Increased EE, low BW, increased insulin sensitivity
Knockout	Increased plasma and fecal bile acids Decrease in liver fibrosis and regeneration	Not relevant	Decreased thermogenic ability Decreased ability to expand subcutaneous fat
Metabolic function	Decreased bile acids synthesis	Decreased bile acids synthesis, increased EE, decreased BW, increased glucose disposal and increased insulin sensitivity	Increased EE, decreased BW, increased glucose disposal, increased insulin sensitivity
Mitogenic function	Mitogenic but less mitogenic in mice compared to FGF19	Highly mitogenic in mice	No mitogenic effect in mice FGF21 ko mice are prone to HCC development

## Preclinical models and mode of actions

### Tissue specific actions of FGF19 and FGF21

The insulin sensitizing, BG and BW lowering effects of FGF21 are lost when co-receptor KLB is globally deleted (88). Nevertheless, the global KLB ko mice are surprisingly resistant toward HFD-induced obesity (89). However, KLB is, as described, also a co-receptor for the FGF19/FGF15 system, and therefore mice lacking FGF15 activity have increased plasma bile acids (24, 90). The high plasma bile acids found in the global KLB ko mice may increase EE by activation of G-protein-coupled bile acids receptor (TGR5), which increases EE and GLP-1 release (89). Therefore, tissue-specific silencing of KLB is required to study the contribution of KLB-expressing tissues to the metabolic actions of FGF21. Disruption of KLB, using the Calcium/calmodulin-dependent protein kinase type II subunit alpha (Camk2a) Cre recombinase expressed in neurons, abolishes the beneficial effects of FGF19 and FGF21 on BW loss, glucose, and insulin levels (91). However, KLB in the adipose tissue has been shown to contribute to the insulin-sensitizing effect of FGF19 and FGF21 (88, 91) and mice lacking adipose tissue and mice with adipose-specific deletion of FGFR1 are moreover, refractory to the metabolic benefit of FGF21 (92, 93). FGF19 requires KLB expression in the liver to regulate *Cyp7a1* expression in mice (91), while the positive effect of FGF19 and FGF21 on hepatic steatosis was unaffected by adipose and liver specific KLB deletion (91). It is therefore, clear that activation of the receptor complex in the CNS is required for metabolic activity, but unclear if FGF21 has any direct effect on hepatocytes that contributes to amelioration of NASH.

#### Liver Phenotype in FGF21 Knockout Mice

In response to fasting (94), ketogenic diet (71), high fat diet (95), alcohol (96), and protein restriction (97) the FGF21 ko mice develop liver steatosis. Furthermore, liver weight is already increased in the basal state in the FGF21 ko mice (98). This indicate that FGF21 plays an important role in maintenance of hepatic lipid metabolism. The accumulation of hepatic fat in the FGF21 ko may be linked to an increased flux of FFA from the adipose tissue (98) but may also be caused by a reduction in hepatic β-oxidation due to higher plasma insulin (99) and reduced TG uptake (65) and storage in the adipose tissue. Lack of FGF21 also reduces hepatic FA oxidation in ko mice fed a methionine-choline deficient (MCD) diet which is accompanied with more severe steatosis, peroxidative damage, inflammation, endoplasmic reticulum stress, and fibrosis when compared to wild-type mice (100). FGF21 ko mice are, furthermore, very sensitive to LPS-induced (101) and acetaminophen (APAP)-induced hepatotoxicity compared to wild-type littermates. Finally, FGF21 seems to protect against HCC development, as FGF21 ko mice are found more prone to develop HCC when fed a long term obesogenic diet (95) and mice overexpressing FGF21 are protected toward DEN-induced liver tumors (102).

#### Liver phenotype in FGF15 Knockout mice

Ablation of the *Fgf15* gene in mice increases hepatic *Cyp7a1* mRNA expression the total bile acids pool and faecal bile acids (32). Due to increase bile acids, colon tumour carcinomas are commonly observed in FGF15-deficient mice (103). FGF15 ko mice are like FGF21 ko mice more susceptible to APAP-induced liver injury (104). Interestingly, FGF15 ko mice fed a high fat diet have decreased liver fibrosis while lack of FGF15 had no effect on the severity of liver steatosis or inflammation (105). FGF15 ko mice display an impairment in liver regeneration after partial hepatectomy (48) and have less and smaller tumours and fewer histological neoplastic lesions in response to DEN-induced HCC (49). On the contrary, FGF15 tg mice has increased hepatocyte proliferation suggesting that FGF15 plays a critical role in liver regeneration (50). Other authors, however, claim that in contrast to FGF19, FGF15 does not induce HCC in mice as previously discussed (51).

Taken together, worsening of fibrosis and even earlier development of pre-stage HCC are seen in the mice lacking FGF21. The opposite has been observed in mice lacking FGF15 where a decrease in fibrosis (105) and a decrease in progression to HCC is observed (49). This is distinct from the actions observed by pharmacological dosing, where a decrease in fibrosis is observed in response to both FGF21 (106) and a FGF19 variant (107). FGF19 is however, also a strong inducer of liver carcinomas in mice (108) and it is of high importance to mitigate the mitogenic, FGFR4-mediated effect of FGF19 to allow human therapy, even though species difference may indicate that FGF19 is less mitogenic in human cellular systems (109, 110).

### Pre-clinical Effects of FGF19 and FGF21 and Analogues Thereof in Murine Models of NASH

Predictive pre-clinical models are essential to early drug discovery and with several clinical failures the predictive value of mouse NASH models must be carefully considered. Within NASH several murine models are commonly being used. The models can be divided into 1) dietary/metabolic models like high fructose, high fat, high cholesterol fed mice (DIO NASH) and mechanistic models deficient of essential amino acids like the MCD and choline-deficient (CDA) model; 2) chemical-induced mouse models like streptozotocin for diabetes, carbon tetrachloride (CCl_4_) (liver toxicity), and DEN-induced models for hepatocarcinogenesis (111). Overall, the more metabolic models develop mild inflammation and fibrosis, whilst the mechanistic and toxin-induced models need to be used in a very hypothesis-driven approach with regards to inflammation and fibrosis as they lack most metabolic aspects and are thereby not representative for NASH, but rather a tool to study inflammation and fibrosis in the liver.

The main features of NASH pathology in metabolic models, i.e., steatosis, mild inflammation, and mild fibrosis have all been found to be improved by treatments with FGF21, as well as FGF19 and analogues thereof (86, 87, 107, 112) which may partly be driven by a decrease in BW. However, in the lipotoxic mechanistic models (MCD, CDA-HFD) with a higher degree of inflammation and fibrosis, FGF21 treatment improved all parameters of importance in NASH, without lowering BW in the MCD model (97, 106, 112, 113). Interestingly, an improvement in steatosis was observed in response to FGF21 although the deficiency to hepatic lipids in these models often limits treatment effects on steatosis. The effect of FGF21 on steatosis in the MCD and CDA-HFD models may be linked to FGF21’s ability to increase FA oxidation or to decrease DNL (97, 113). However, it has to be stressed that the MCD model is a very harsh model with significant BW loss induced by the diet. Therefore, the CDA-HFD fed mice which are more BW stable is the preferred mechanistic model of the two (114).

In addition to the anti-inflammatory and anti-fibrotic effects of FGF21 and FGF19 in the metabolic as well as more mechanistic models of liver disease, several studies support a role in resolving fibrosis independent of BW loss. Administration of FGF21 improves inflammation and fibrosis in diabetic nephropathy (115, 116), pulmonary fibrosis induced by bleomycin (115), cardiac fibrosis (117, 118), as well as pancreatic fibrosis (119). FGF19 has also shown beneficial effects in diabetic cardiomyopathy improving both cardiac function and decreasing fibrosis (120). Finally, FGF21 has been shown to attenuate dimethylnitrosamine (DMN)-induced hepatic fibrogenesis in mice by inhibition of hepatic stellate cells (HSC) activation *via* down-regulating the expression of transforming growth factor (TGF)β (121).

In summary, both FGF21 and FGF19 analogues decrease steatosis, inflammation and fibrosis in various NASH models. Furthermore, FGF21 prevents fibrosis in numerous tissues (lung, heart, and pancreas) in mice, while less data is available for FGF19.

### Mode of Action

#### Anti-Steatotic Effects

Reversal of hepatic steatosis is of crucial importance to improve liver health and several pharmacological approaches to lower DNL or to increase lipid oxidation are in development (17). FGF19 and FGF21 both depend on KLB expression in the CNS to lower hepatic steatosis (91). The effect on steatosis is likely independent of the FGFR4/KLB complex as a FGF19 variant lacking FGFR1c/KLB activity lack metabolic activity (24, 30). Both FGF21 (64) and FGF19 (79) increase EE in mice causing BW loss by inducing corticotropin-releasing factor (CRF) and sympathetic nerve activity (122) but beside that, there are several means by which FGF19 and FGF21 decrease steatosis. First of all, FGF21 has been described to inhibit lipolysis from the adipose tissue (60, 123) preventing flux of FFA to accumulate in the liver. Furthermore, FGF21 has been shown to increase TG uptake in the adipose tissue by induction of LPL’ase activity (65). A decrease in the delivery of triglyceride-enriched very low-density lipoprotein (VLDL) to the liver by downregulating VLDL receptor expression has also been described as a mechanism by which FGF21 treatment lowers hepatic steatosis (124) (125). Interestingly, FGF21 has also been shown to increase hydrogen sulfide (H2S) (125) which is a potent stimulator of autophagic flux which plays an important role in liver triglyceride clearance (126).

Another important contributor to the observed improvement in steatosis is the reduction in plasma insulin which decreases *de novo* lipogenesis by lowering sterol regulatory element-binding protein 1 (SREBP-1) activity (127) and increases beta-oxidation (59, 86, 87). Inhibition of hepatic mTOR by FGF21 (128, 129) may also be part of the lipid lowering mechanism as mTOR is a major regulator of lipid metabolism (130) and likely contribute to the effect of FGF19 and FGF21 on hepatic lipid metabolism. It is still not fully understood if a direct action on hepatocytes contribute to the positive effect of FGF19 and FGF21 on steatosis, but overexpression of an inactive KLB mutant interestingly induces intracellular lipid accumulation in HepG2 and Huh7 cells *in vitro* (131).

#### Regulation of Oxidative Stress and Autophagy

It is well described that metabolic stress in hepatocytes, as induced by excess FFA, free cholesterol, and TG, will lead to increased reactive oxygen species (ROS), endoplasmic reticulum (ER) stress and oxidative stress, as well as impaired autophagy (132). When the antioxidant capacity of the hepatocytes is surpassed, DNA damage and oxidation occur, eventually resulting in cell death, either *via* apoptosis or necroptosis, which in turn triggers hepatic inflammation.

The MCD model, treated with FGF21 have enhanced hepatic mitochondrial function which has been shown to attenuate hepatic ER stress (112). Both FGF21 (120) and FGF19 (133) have furthermore in heart and liver, respectively, been shown to active the nuclear factor erythroid-2 related factor 2 (Nrf2) pathway. Activation of Nrf2 increases the expression of antioxidant proteins which protects the cells toward oxidative damage. FGF21 has been shown to activate AMP-activated protein kinase (AMPK) in adipocytes (134) and hepatocytes (135) which prevents hepatocyte apoptosis (136) and reduces ER-stress in NASH (137). Likewise, FGF19 has been shown to activate the AMPK pathway and promote antioxidant response in muscle and heart (120). It is still not fully understood if FGF21 activates AMPK through direct effects on hepatocytes, but interestingly adiponectin which is induced by FGF21 in several species (64, 138–140) is an activator of AMPK release (141). Adiponectin ko mice are also refractory to increase insulin sensitivity in response to FGF21 treatment (142). More data are needed to understand if the beneficial effect of FGF21 on NASH is dependent of adiponectin. It is not clear if FGF19 also increases the expression of adiponectin as one study shows that FGF19 does not increase plasma adiponectin (91), while another study found that mice deficient in FGF15 have lower adiponectin levels (143).

#### Anti-Inflammatory Effects

The anti-inflammatory effect following administration of a FGF21 analogue has been shown to be mediated *via* inhibition of interleukin (IL)-17A expression in pro-inflammatory T helper 17 (Th17) (113) and the effect seems to be mediated *via* increases in adiponectin (113). Furthermore, in *ob/ob* mice, FGF21 treatment reduces the phosphorylation of hepatic nuclear factor kappa B (NF-kB), the main inflammatory signaling pathway activated by proinflammatory cytokines, which also indicate an anti-inflammatory action of FGF21 (144). Interestingly, NF-kB is also a downstream target of AMPK activation (145). Moreover, as FGF21 has been shown to increase the HPA axis in mice (122, 146) an increase in plasma corticosterone may also contribute to the anti-inflammatory effect.

#### Anti-Fibrotic Effects

During liver injury, HSCs become activated and trans-differentiate into myofibroblasts. The effect is mediated by connective tissue growth factor (CTGF) and Transforming growth factor beta (TGFβ) which increase proliferation and fibrogenesis augmented by inflammation and immunoregulation, as well as altered matrix degradation. Oxidative stress is one of the important drivers of fibrogenesis through activation of TGFβ in several pathological conditions (147). FGF21 and FGF19 may exert anti-fibrotic effects by resolving lipotoxicity and activating the oxidative stress defence as described above. Whether some of the anti-fibrotic effects of FGF21 are mediated *via* adiponectin actions cannot be excluded (148). Furthermore, the reduced bile acids toxicity is believed to play a role in the FXR- and FGF19-mediated anti-fibrotic effect (107).

Surprisingly, direct anti-fibrotic actions by FGF19 and FGF21 have been described in human LX-2 cells (106, 149, 150). It is, however, unclear if these myofibroblast express KLB and more data required to understand if FGF19 and FGF21 act direct on HSC. *In vivo*, FGF21 has been shown to decrease the expressions of G-protein coupled receptor (GPR)91 and markers of fibrosis (alpha-smooth muscle actin (α-SMA) and collagen type 1) in the liver of MCD fed mice (106) but it is unknown if this is mediated by a direct effect in the liver. Finally, FGF21 is upregulated in response to protein restriction (68) and downstream actions of FGF21 may therefore also involve regulation of protein synthesis and catabolism which may affect the novo synthesis of collagen, but more data are required to support this hypothesis. FGF15/FGF19 has on the other hand been shown to increase hepatic protein synthesis (44).

#### Regulation of Bile Acids

Bile acids are toxic (41) and are tightly regulated by several mechanisms and excessive amount of bile acids is known to cause liver damage (41). The decrease in bile acids synthesis observed in response to NGM282 and FXR agonist treatment have a beneficial effect on the liver especially in cholestatic liver diseases (151, 152). Therefore, FGF19 may also promote liver health by reducing the bile acids levels in NASH (49, 153). The effect of FGF21 in bile acids metabolism is less well described but supraphysiological doses of FGF21 may interact with the FGFR4/KLB system and FGF21 has been shown to decrease Cyp7A1 and bile acids in pre-clinical species (83).

#### Regulation of Plasma Lipids

In contrast to FGF19 and analogues thereof, which increase plasma cholesterol and TG (28, 85) FGF21 lowers plasma cholesterol and TG (86, 87). Based on receptor specific FGF19 analogues it is well established that the negative impact on plasma cholesterol and TG by FGF19 treatment is mediated *via* FGFR4/KLB activation (85). Inhibition of Cyp7a1 decreases bile acids synthesis from cholesterol hence plasma cholesterol is likely to increase. The increase in plasma TG induced by FGF19 in mice (28, 85) may be linked to a decrease in FXR activity as FXR KO mice have increased plasma TG (152) but the high plasma cholesterol may also activate hepatic liver x receptor (LXR)a causing an increase in plasma TG (154). The positive effect of FGF21 on plasma lipids is mediated by FGFR1c/KLB (91) and as FGF19 can activate both FGFR1c/KLB and FGFR4/KLB the effect of FGF19 on plasma lipid is a mixture of FGFR1/KLB lipid lowering effect and the negative impact of FGFR4/KLB activation on plasma lipids.

#### Summary

FGF21 clearly lowers hepatic steatosis in mice and multiple mechanisms ranging from decreases in BW, increases in beta-oxidation to increases in autophagy may play important roles. The mode of action behind the anti-inflammatory and anti-fibrotic effects are less elucidated. A strong increase in adiponectin may link the positive effect of FGF21 to the observed anti-inflammatory and anti-fibrotic effects. As FGF19 also activates the FGFR1c/KLB receptor complex (28) and requires CNS receptor action for metabolic activity (91) it is likely that FGF19 resembles FGF21 in the regulation of adiponectin. The contribution of bile acids lowering to the anti-fibrotic action is another topic of interest and therefore the effect of FGFR4-selective FGF19 analogues on NASH outcome in mice will be of great interest. Increased knowledge of the receptor complex expression in healthy and diseased murine and human liver cells (hepatocytes, immune cells, and myofibroblasts) with validated cell specific markers will furthermore help elucidate if direct effect of FGF19 or FGF21 on immune and/or HSC can be expected.

## Clinical Findings

### Regulation of Endogenous FGF19 and FGF21 in NASH

Plasma FGF21 is mainly liver derived (155) and is as described previously regulated by high FFA (57–59), high glucose (61) and lack of amino acids (68). Plasma FGF21 displays a circadian regulation with peak levels around 3-6 am (156, 157). Plasma FGF21 is positively correlated to BMI and insulin resistance in humans (158–162) which has resulted in discussion of FGF21 resistance (163). However, other authors found no evidence of FGF21 resistance in obese mice (164) and as described below FGF21 analogues are able to lower BW, plasma lipids and improve insulin sensitivity in obese humans indicating lack of overt FGF21 resistance in obese humans (165–167). Furthermore, liver fat is also positively correlated to plasma FGF21 (168, 169) and plasma FGF21 is increased in patients with NAFLD (170–173) and NASH (158, 174–176). Liver fat is the strongest BMI-independent marker of hepatic FGF21 expression and plasma FGF21 (174, 175). FGF21 has, therefore, been suggested to be a potential diagnostic biomarker of NAFLD (177). It is, however, important to note that fibroblast activating protein (Fap) which has been found to inactivate FGF21 (178) is increased in NASH patients (179) and future studies are needed to distinguish between total and active plasma FGF21 in NASH. The increase in plasma FGF21 in response to metabolic impairment and NAFLD may represent an adaptive protective response where increases in FGF21 may act to increase insulin sensitivity and decrease liver fat. Reduction of liver fat by tesamorelin treatment in HIV patients or by GLP-1 receptor agonist treatment in T2D lead to reductions in liver fat content which has been associated with a decrease in plasma FGF21. The regulation of plasma FGF21 in health and disease has recently nicely been reviewed by Keuper, et al (180).

FGF19 is expressed in the intestinal enterocytes and is as described increased in response to bile acids by activation of the FXR (181). FGF19 displays a diurnal rhythm with two major peaks at 3 and 9 pm (182). Fasting FGF19 has been shown to be increased in response to bariatric surgery (183, 184) where bile acids are known to be increased (185). Opposite to plasma FGF21, fasted serum FGF19 levels are reduced in individuals with overweight, obesity (186) and NAFLD (187, 188). FGF19 has therefore also been suggested as a diagnostic biomarker in NASH where a decrease should indicate increases in steatosis (187). The reduced serum FGF19 levels in children with NASH is, however, not statistically associated with paediatric NAFLD histological score (187, 189). Furthermore, hepatic response to FGF19 seemed to be impaired in humans with NAFLD (190) and lack of FGF19 and decreased FGF19 activity may worsening NASH due to accumulation of toxic bile acids. As expected, serum FGF19 correlates with severity of cholestatic liver disease (191) where increases in serum FGF19 is associated with a decrease in CYP7A1 expression (191). The differential regulation of plasma FGF21 and FGF19 in humans further support distinct physiological roles of the two endocrine FGFs. Plasma FGF21 is increased in NASH while FGF19 seem to be downregulated, thus it is of high interest that pharmacological intervention with analogues of the two hormones improves NASH resolution and decrease fibrosis in humans as described below.

### Genetic Evidence

Human genetics are important to understand the relevance of a given gene in a specific disease. Within the last few years polymorphisms in the FGF21 and KLB gene have revealed important phenotypic information supporting findings in gene modified animal models.

#### Polymorphisms in FGF21

Two independent studies in humans have shown that single nucleotide polymorphism (SNPs) in the FGF21 locus are associated with changes in intake of macronutrients. The two alleles rs838133 and rs838145 are both associated with higher carbohydrate intake and therefore, potentially, also with a loss of FGF21 function. In the Danish Inter99 cohort, rs838133 was furthermore linked to an increased consumption of candy and decreased fat and protein intake (192, 193). The effect of FGF21 on food preference was later confirmed in a meta-analysis including up to 123,000 individuals (194). A GWAS from the UK Biobank (>450,000 individuals) showed that the common rs838133 allele also is associated with insulin resistance, higher blood pressure (PB) and a higher waist-to-hip ratio despite a lower total body-fat percentage (195). Nevertheless, the effect of the rs838133 allele on these parameters is extremely small (0.33 mm Hg in PB and a 1 mm difference in hip circumference), but the effect sizes of common genetic variants does not always predict the potential efficacy of a target in response to pharmacological intervention. Notably, subjects with a high hip-to-waist ratio have low plasma adiponectin (196) and subjects with high hip-to-waist ratio are also prone to develop NASH (197, 198). The inverse correlation between adiponectin and fasting insulin, HOMA-IR, triglyceride, systolic and diastolic BP (196, 199) potentially links adiponectin to FGF21 biology (142). The FGF21 rs838133 allele is, however, not associated with fasting plasma glucose, but is interestingly, associated with higher plasma low-density lipoprotein cholesterol (LDLc) and higher gamma-glutamyl transpeptidase (GGT) levels (195).

#### Polymorphisms in FGF19

FGF19 loss of function in humans is expected to increase bile acids to a toxic level and we have not been able to identify any GWAS on FGF19 loss of function. Two common SNPs (rs948992 and rs1789170) in the FGF19 gene were not found to be associated with bile acids diarrhea (BAD) (200), however, reduced plasma FGF19 levels have been correlated to BAD (201). On the other hand, increased FGF19 copy number is frequently detected in HCC (202).

#### Polymorphisms in KLB

Loss of KLB function will of course affect both FGF19 and FGF21 activity in humans. A SNP (rs17618244) in KLB has been associated with colonic transit in patients with diarrhea-predominant irritable bowel (203) which presumably is due to change in bile acids metabolism caused by a decrease in FGF19 activity. Furthermore, in a meta-analysis including more than 105,000 individuals, a locus in KLB was associated with increased alcohol consumption (204). A common SNP in the KLB gene (rs2608819) has also been associated with a reduction of KLB expression in the adipose tissue and a higher body mass index (BMI) potentially linking FGF21 activity to EE in humans (205). It is therefore, of interest to note that rs17618244 SNP is associated with increased risk of ballooning and lobular inflammation in children with NAFLD (131). It is, however, unknown if loss of KLB function prone children to NASH due to lack of FGF19 or FGF21 activity or both.

### FGF19 and FGF21 Analogues in Clinical Development for NASH

#### FGF19 Analogues

While a handful of FXR agonist are in late stage clinical trials (17) there is currently only one FGF19 analogue in clinical development and as described previously it is important to separate the mitogenic signaling from the metabolic action of FGF19.

##### Aldafermin

NGM282 (Aldafermin) is a non-mitogenic FGF19 analogue with 5-amino acid deletion (P24-S28) and 3 amino acids substitutions at critical positions (A30S, G31S, H33L) within the amino terminus (183, 206). The analogue is not protracted, and once daily subcutaneous dosing is required. These mutations prevent Aldafermin to activate signal transducer and activator of transcription 3 (STAT3), a signaling pathway essential for FGF19-mediated HCC, while Aldafermin retains its ability to inhibit CYP7A1 (107, 110, 151, 207). Aldafermin is thereby designed to be non-mitogenic and does not induce liver proliferation in mice (208). Aldafermin decreases serum levels of 7α-hydroxy-4-cholesteb-3-one (C4) by inhibition of hepatic CYP7A1 transcription in humans (151). In a randomized, double-blind, placebo-controlled study in patients with type 2 diabetes 2, 5, 10 mg NGM282 (sc injection once daily for 12 weeks) did not correct hyperglycemia while a significant improvement in insulin sensitivity was observed at the high dose at the end of the study (183). In a phase 2 trial in patients with primary biliary cholangitis, a devastating liver disease caused by hepatic accumulation of toxic bile acids (209), once daily administration of Aldafermin for 28 days lowered plasma bile acids and improved liver function (210). Furthermore, in a phase 2 study in patients with NASH 12 weeks of Aldafermin treatment reduced absolute liver fat by 5% measured by magnetic resonance imaging proton density fat fraction (MRI-PDFF) in 80% of the patients (207). A significant decrease in plasma liver enzymes alanine aminotransferase (ALT) and aspartate amino transferase (AST) was also observed in response to Aldafermin treatment (207). Furthermore, plasma C4 was decreased by more than 95% within the first day of treatment and a significant increase in plasma LDLc was observed while plasma TG was decreased (207). A significant decrease in BW was observed in the highest dose group (207). Co-administration with statins was later shown to be able to normalize the Aldafermin-induced increases in plasma LDLc (211). Aldafermin also improves histological endpoints after 12 weeks of treatment in patients with biopsy-confirmed NASH (212). Of the 43 patients who received subcutaneous Aldafermin (1 mg, n=24; 3 mg, n=19) once daily for 12 weeks a significant improvement in NAS score by 2 or more points without worsening of fibrosis was observed in more than 50% of the patients. Furthermore, liver fibrosis was improved by one stage or more without worsening of NASH in 25% and 41% of patients who received Aldafermin 1 or 3 mg, respectively (212). Aldafermin, furthermore, reduced pro-peptide type III collagen (Pro-C3), a biomarker of fibrogenesis (140, 213), in plasma with 22% and 33% in response to 1 and 3 mg respectively (212). However, no placebo group was included in the trial and the significance of these effect needs to be confirmed. The data was recently confirmed in a 24 weeks trial (78 patients with F2/F3) where fibrosis improvement (>1) and no worsening of NASH was observed in 38% of patients treated with Aldafermin versus only 18% in the placebo group (214). NASH resolution with no worsening of fibrosis was observed in 24% of patients receiving Aldafermin compared to 9% in the placebo group (214). It is therefore, of interest to note that the effect of OCA, which is a upstream regulator of FGF19, had limited on the regulatory endpoints in the phase 3 trial (REGENERATE), but the endogenous levels of FGF19 induced by OCA (215) may not be high enough to induce the metabolic response mediated by FGFR1c/KLB interaction.

##### Adverse Effects

Aldafermin is in general well tolerated, but dosing of Aldafermin is associated with dose-related abdominal cramping and diarrhea (207, 210, 212, 216). Approximately, 10% of the patients receiving Aldafermin were discontinued due to gastrointestinal (GI) side effect such as high frequencies of diarrhea, abdominal pain and nausea. In a follow up study Aldafermin was shown to alter bowel function and accelerates gastric and colonic transit (216) which is likely caused by changes in bile acids metabolism. Furthermore, 14% of subjects dosed with 3 mg Aldafermin reported an increase in appetite (216) similar to observations in clinical trial with FGF21 analogues (166, 217, 218), thus overlapping effects on regulation of appetite may appear. The increase in LDLc is furthermore, a major concern as most patients with NASH have an increased risk of cardiovascular diseases (7, 219) and hence counterregulatory treatment with, i.e., statins is required.

#### FGF21 Analogues

Native FGF21 has a short half-life (t½) (220) and analogues with protracted action have been designed. A variety of approaches (polyethylene glycol-modified (pegylation) (221–224), Fc-fusions (225–227) and immunoglobulin-fusion (228) have been applied to increase the half-life. The N- and C-terminals of FGF21 are furthermore important to maintain potency (229, 230) and FGF21 analogues with stabilized N- and C-terminal have been designed (231, 232). In this review we only include the two most advanced FGF21 analogues (Pegbelfermin and Efruxifermin) as these have clinical data in NASH. For more specific review of other FGF21 analogues see (233).

##### Pegbelfermin

Pegbelfermin is a PEGylated FGF21 analogue (224), however, no amino acids are substituted in the C-terminal to protect toward C-terminal degradation (82). In a double blinded, placebo controlled study in obese patients with T2DM an increase in plasma high-density lipoprotein cholesterol (HDLc) and a decrease in plasma TG was observed in response to Pegbelfermin treatment (1, 5, or 20 mg once daily or 20 mg once weekly for 12 weeks) while no effect on glycemic control or BW was observed (167). A dose dependent increase in plasma adiponectin was observed (167). In a Phase 2 clinical trial, 16 weeks of Pegbelfermin treatment (10 mg once daily or 20 mg once weekly) decreased absolute hepatic lipid content by 6.8% measured by MRI-PDFF in the 10 mg once daily group while 20 mg once weekly induced a decrease of 5.2% compared to placebo (140). As observed in the phase 1b trial, Pegbelfermin increased plasma adiponectin and HDLc while fasting plasma LDLc and TG were decreased (140). Liver stiffness, measured by magnetic resonance elastography (MRE), was also decreased as well as plasma Pro-C3. Currently, two clinical phase 2b trials (NCT03486899 and NCT03486912) of 24- and 48-weeks duration are ongoing in patient with NASH F2-F3 and F4, respectively and the result is expected to support further development of Pegbelfermin for the treatment of NASH.

##### Efruxifermin

Efruxifermin is a FcFGF21 analogue with N- and C-terminal modification to prevent degradation and increase potency (82). In mice as well as monkeys FcFGF21RG outperform native FGF21 (82). The t½ of Efruxifermin is 2-4 days in humans supporting a once weekly dosing. In a Phase 1b trial of 4 weeks duration 7–140 mg of Efruxifermin lowered plasma TG and LDLc and increased HDLc. Postprandial decrease in FFA was also observed in subjects treated with Efruxifermin (206). Efruxifermin also lowered BG and glycosylated HbA1c and an increase in insulin sensitivity was observed at the 70 mg once weekly dosing. As seen for other FGF21 analogues, a dose-dependent increase in adiponectin was observed. Recently, data from a phase 2 study in patients with biopsy confirmed NASH has been published (www.akerotx.com). In response to 12 weeks of Efruxifermin treatment (28, 50, and 70 mg once weekly) liver steatosis was reduced up to 70% (MRI-PDFF) in all patients. No significant dose response was observed indicating that the tested doses of 27, 50, and 70 mg once weekly were on the upper flat curve of the dose response. Patients with more than 30% reduction in liver fat were eligible for a liver biopsy post treatment and thus, unfortunately, only a couple of biopsies were taken from the placebo treated subjects. Nevertheless, a significant effect on NASH resolution and a decrease in fibrosis of >1 stage was observed in 39% of the subjects treated with 50 mg Efruxifermin. A dose dependent increase in plasma adiponectin was observed in all dose levels. The pronounced and significant effect of Efruxifermin make this compound a promising treatment option for NASH and recently Efruxifermin obtained European Medicines Agency Priority Medicines (PRIME) Designation in NASH.

##### Adverse Effects

Both Pegbelfermin and Efruxifermin are well tolerated. The most common side effect is GI related with increased frequency of diarrhea and nausea, but also an increase in appetite have been reported (140, 206). Interestingly, incidence of diarrhea is increasing with increasing doses which may indicate an interaction with bile acids synthesis and thereby FGFR4/KLB activity. Finally, anti-drug antibodies (ADA) will have to be carefully evaluated as, e.g., Pegbelfermin induces ADA, which may cross-react with the endogenous FGF21.

The FGF21 tg mice have reduced female fertility (25), increased plasma corticosterone (76) and lower bone mineral density (234). In the clinical setting bone markers have been shown to change in response to treatment with an FGF21 analogue (PF05231023) in obese subjects, however a decrease in BW was also observed in response to PF05231023 (166, 235) and therefore, it is impossible to conclude whether the change in plasma bone marker was related to FGF21 treatment or BW loss per se (236). Moreover, no apparent effect on bone density (assessed by bone densitometry) was observed in patients receiving Pegbelfermin for 16 weeks (140). However, based on previous data with PAPRγ agonists (Thiazolidinediones) which decrease BMD (237), future studies of longer duration are required to understand the impact of FGF21 on bone health in humans. The negative effect on female fertility of FGF21 also needs to be closely monitored but in the lean tg mice the adverse effect of FGF21 on fertility may be linked to lack of energy due to a large decrease in BW (86). It is well established that low leptin decreases fertility (238) and in the tg mice the decrease in fertility cause by FGF21 can be overcome by feeding the mice a high fat diet (239). As FGF21 have overlapping activities with FGF21 in mice by activation of the FGFR1c/KLB complex (28), it is of great interest to understand if similar adverse findings were observed in rodents or NHPs treated with Aldafermin.

### Overlapping and Distinct Effect of FGF19 and FGF21 in Humans

A summary of the pharmacological effects of FGF21 and FGF19 analogues in humans is shown in **Table 2**, highlighting that both FGF19 and FGF21 analogues lower hepatic steatosis and fibrotic biomarkers in humans. However, differential effect on plasma cholesterol is observed. The FGF21 analogues have strong effect on the FGFR1c/KLB complex and potential also a slight effect on the FGFR4/KLB complex, however, as plasma C4 and total bile acids have not been measured in response to FGF21 treatment in humans, it is not possible to conclude on this. As FGF19 also binds with high potency to the human FGFR1c/KLB complex overlapping effect with FGF21 analogues is expected. As adiponectin may be involved in the anti-inflammatory and anti-fibrotic actions of FGF21 it will be of interest to understand if also Aldafermin increases plasma adiponectin in humans. Future clinical studies are required to determine which approach is more beneficial for patients with NASH and if blockage of bile acids synthesis, which may increase plasma LDLc and increase bowel movement, is advantageous in NASH and hence acceptable.

**Table 2:** Summary of observations in clinical trials of FGF19 and FGF21 analogues.

Compound	Aldafermin	Pegbelfermin	Efruxifermin
**Blood glucose**	↔	↔ ↓	↓
**Body weight**	↔	↔	↓
**Insulin sensitivity**	↔	↑	↑
**Hepatic steatosis**	↓	↓	↓
**Hepatic fibrosis**	↓	↓	↓
**Serum Pro-C3**	↓	↓	↓
**Plasma TG**	↓	↓	↓
**Plasma cholesterol**	↑	↓	↓
**Serum C4**	↓	ND	ND
**Plasma adiponectin**	ND	↑	↑

ND, not determined.

### Conclusion

**Figure 3:**
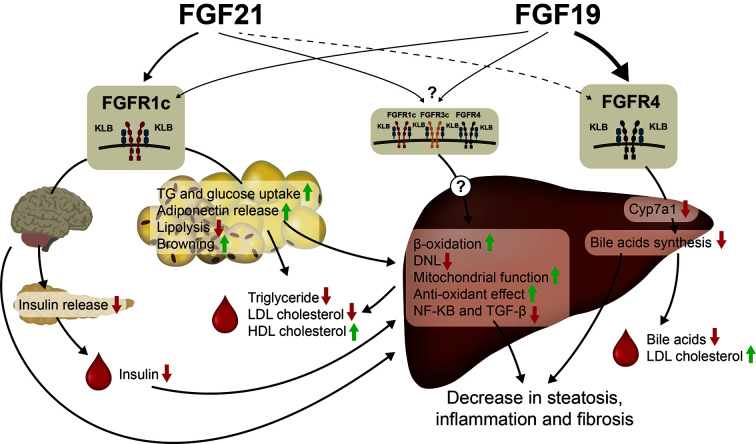
FGF19 and FGF21 treatment of NASH. The effect on hepatic steatosis, inflammation and fibrosis seem to be mediated *via* activation of the FGFR1c/KLB complex in the CNS and in the adipose tissue. FGF21 has been shown to decrease insulin release which will increase hepatic beta-oxidation and decrease DNL. FGF21 is furthermore a strong inducer of adiponectin release which has been shown to have several beneficial effects on NASH. FGF21 also increases the antioxidant capacity of the liver and increase the mitochondrial function. In addition, FGF21 lowers plasma TG, LDLc, and increases plasma HDLc. FGF19 has also been shown to activate the FGFR1c/KLB pathway but in addition FGF19 decreases bile acids synthesis *via* FGFR4/KLB activation, which has beneficial effect on NASH. It is unknown, if FGF21 activates the FGFR4/KLB complex and if other FGFRs (FGFR2c and FGFR3c) expressed in liver are involved in direct action of FGF19 or FGF21.

FGF19 and FGF21 analogues have overlapping effect on steatosis, inflammation and fibrosis in mice and human subjects. The suggested mode of action studied in pre-clinical models are therefore likely also presented in humans emphasizing that BW loss is not the major driver of NASH resolution and decrease in fibrosis. Whether the effects are direct or indirect actions on the liver is still to be confirmed. However, while FGF21 analogues lower plasma lipids, FGF19, and Aldafermin have been shown to increase plasma cholesterol and decrease plasma bile acids in mouse and human. The beneficial effect on NASH is likely mediated by the FGFR1c/KLB complex, while the contribution of the FGFR4/KLB complex and lowering of bile acids preventing hepatocyte damage and subsequent fibrosis is not fully established in NASH. The results from phase 2b trials (e.g., NCT04171765) where administration of FGFR1c/KLB specific antibodies (240) are subjected to NASH patients will reveal more details on the contribution from the FGFR4/KLB complex to NASH resolution and lowering of fibrosis. It is furthermore, to be established if Aldafermin is non-mitogenic or even protective toward HCC by inhibiting actions of endogenous FGF19 in humans. FGF21 has been shown to protect toward development of HCC in mice and long-term outcome studies are required to show a decrease in HCC progression of potentially both Aldafermin and FGF21 analogues. Future research questions related to FGF19 and FGF21 within the NASH field are summarize in **Table 3**. The metabolic effects of FGF19 and FGF21 is summarized in **Figure 3**. In conclusion, FGF19 and FGF21 analogues have significant effect on NASH resolution and fibrosis in small, short term clinical trials. Thus, much is to expect of these classes of compounds for future treatment of NASH if long term safety is acceptable.

**Table 3:** Future research questions for FGF19 and FGF21 with the NASH field.

Why does FGF15 and FGF19 display differential receptor selectivity?
Dissect the contribution of direct actions of FGF21 and FGF19 on the liver in NASH resolution and fibrosis: In which cells are FGF receptor subtypes co-expressed with KLB in healthy and diseased livers? What is the impact of FGFR4/KLB mediated effects on NASH resolution and fibrosis vs FGFR1c/KLB mediated effects (effect of FGFR4 vs FGFR1c specific FGF19 analogues in pre-clinical models)? What is the contribution of FGFR2c/KLB and FGFR3c/KLB activation by FGF19/FGF21 in NASH? Are the beta KLB CNS and adipose tissue specific ko models more prone to develop NASH?
What is the contribution of increased adiponectin levels, induced by FGF21 and potentially FGF19 therapies, on NASH resolution and fibrosis?
Does FGF21 analogues regulated bile acid metabolismin humans
Are carriers of the FGF21 rs838133 allele at higher risk for development of NASH and/or HCC?

## Author Contributions

EH and BA contributed equally to this review. All authors contributed to the article and approved the submitted version.

## Conflict of Interest

EH and BA are employees and minor stakeholders of Novo Nordisk A/S.
